# Hsd17b7 undergoes dynamic subcellular localization during Neuro2a differentiation

**DOI:** 10.3389/fnmol.2025.1639803

**Published:** 2026-01-15

**Authors:** Matthew Bispo, Macey Pennay, Joe C. Brague, Ashley M. Driver

**Affiliations:** 1 Neuroscience Program, University of Scranton, Scranton, PA, United States; 2 Department of Biology, University of Scranton, Scranton, PA, United States

**Keywords:** cholesterol biosynthesis, Neuro2a, Hsd17b7, neural differentiation, localization

## Abstract

Enzymes within the cholesterol biosynthesis pathway, particularly those in post-squalene biosynthesis, have been linked to abnormal neurodevelopment. Alterations of individual enzymes manifest unique brain phenotypes, suggesting each enzyme has distinct roles within the mammalian neural cell. However, a comprehensive characterization of cholesterol biosynthesis enzymes to understand these differences has yet to be fully obtained. Therefore, this study aimed to contribute to this growing body of knowledge by characterizing the subcellular localization of the cholesterol biosynthesis enzyme Hydroxysteroid-17-beta7 (Hsd17b7) within a mammalian neural cell line. Using mouse Neuro2a cells, we compared expression patterns between both endogenous Hsd17b7 and GFP-tagged constructs. Using confocal microscopy, we noted Hsd17b7 absence in the Golgi and lysosomes while confirming its presence in the endoplasmic reticulum. Of interest, we also observed co-localization with the nuclear membrane, which had not been established. Upon 24-hour serum deprivation, patterns of Hsd17b7-GFP in differentiated cells were still observed in the cell body, as seen in the undifferentiated cells. However, we also observed evidence of GFP-positive protein localization within MAP2-positive neurites. Co-staining with Hsd17b7 antibody and conjugated Phalloidin further supported the localization of Hsd17b7 within developing neurites. Together, this suggests a potential role for Hsd17b7 within early axons and dendrites, however, further investigation is needed to determine potential implications on neural differentiation.

## Introduction

Cholesterol biosynthesis enzymes have had a growing importance in mammalian neural development. In both human and animal models, reduced function of cholesterol biosynthesis enzymes, especially during post-squalene biosynthesis, can result in a variety of neurodevelopmental phenotypes (Herman, 2003; Orth and Bellosta, 2012; Porter and Herman, 2011). It is of particular interest to note that the observed phenotypes vary from lethal embryonic underdevelopment of the brain to milder, survivable structural defects (Porter and Herman, 2011). To gain a better understanding as to why these enzymes manifest into unique phenotypes, characterization of these proteins within mammalian cells is warranted.

A growing body of evidence has been generated on the function and location of various cholesterol biosynthesis enzymes within mammalian cells. Enzymes early in the cholesterol biosynthesis pathway have been shown to localize to peroxisomes in HepG2 cells (Kovacs et al., 2007). In contrast, the post-squalene enzyme NAD(P)-dependent steroid dehydrogenase-like protein (Nsdhl) has been shown to localize to both the ER and lipid droplets (Caldas, 2003). Studies in mouse Neuro2a cells have shown that 24-Dehydrocholesterol reductase (Dhrc24), Emopamil-Binding Protein (EBP), and 7-dehydrocholesterol reductase (Dhcr7) localize to both the endoplasmic reticulum (ER) and nuclear envelope (Koczok et al., 2019). Interestingly, Dhcr7 also showed localization to the Golgi apparatus (Koczok et al., 2019). The diversity in protein location is intriguing given these enzymes appear to have unique cellular impacts during mammalian brain formation.

Neural cells are of particular focus as they have a heavy dependency on cholesterol biosynthesis during mammalian development. Formation of the blood brain barrier partitions developing neurons of the brain from systemic cholesterol stores. Therefore, *de novo* biosynthesis within developing neurons provides a majority of the cholesterol during early neurogenesis (Genaro-Mattos et al., 2019). The dependency on de *novo* synthesis supports that these enzymes are active during early neurogenesis (Fünfschilling et al., 2012). Studies have shown that absence of cholesterol biosynthesis in mammalian neural stem cells increases cell death (Saito et al., 2009) and can alter the process of neurogenesis (Driver et al., 2016). While the subcellular location of select post-squalene biosynthesis enzymes has been reported in mammalian neural cells, a comprehensive picture of all enzymes within the cholesterol biosynthesis pathway remains incomplete.

The primary aim of this study is to characterize the subcellular localization of the cholesterol biosynthesis enzyme Hydroxysteroid17-β7 (Hsd17b7) in mouse Neuro2a cells. This enzyme is a sterol desaturase that functions at two different points in post-squalene biosynthesis. It functions as a 3-ketosteroid reductase converting 4-methylzymosterone to 4α-methylzymosterol and zymosterone to zymosterol (Marijanovic et al., 2003). In addition to its roles in cholesterol biosynthesis, this enzyme is also a steroid dehydrogenase, converting estrone into estradiol and dihydrotestosterone to 3β-androstane-diol (Marijanovic et al., 2003; Shehu et al., 2011). Studies in mouse models have shown that a reduction of Hsd17b7 function results in severe neurodevelopmental phenotypes including significant lack of forebrain development (Shehu et al., 2008; Stottmann et al., 2011). Further research has shown that reduction of Hsd17b7 also promotes precocious differentiation and abnormal morphology in primary mouse neurons (Driver et al., 2016). However, the underlying mechanism for this is not understood.

Given the links between Hsd17b7 and abnormal neurogenesis (Driver et al., 2016), this study focuses on the subcellular localization of Hsd17b7 in both undifferentiated and differentiated Neuro2a cells. We report that while Hsd17b7 does localize to the endoplasmic reticulum, it is also associated with the nuclear membrane. Moreover, localization of this protein appears dynamic during neurogenesis, with Hsd17b7 localizing within the neurites of differentiating Neuro2a cells. This would suggest that processes like cholesterol biosynthesis may be occurring in regions other than the soma, providing new implications on how this enzyme may impact structures such as developing axons and dendrites.

## Materials and methods

### Plasmid procurement and preparation

N-terminal and C-terminal tagged GFP plasmids with full length mouse Hydroxysteroid 17-β7 sequence were obtained from Sino Biological (MG51644-ACG, MG51644-ANG). The pCAG-GFP vector was acquired from Addgene (this plasmid was a gift from Connie Cepko, plasmid # 11150; http://n2t.net/addgene:11150). Hsd17b7 expression plasmids were reconstituted and transformed into OneShot TOP10 chemically competent *E. coli* (C404010, Invitrogen) using manufacturers protocols. Cells were then plated on LB agar plates containing Kanamycin (50 μg/mL). The pCAG-GFP plasmid was obtained as a bacterial stab that was streaked on to an LB agar plate containing Ampicillin (100 μg/mL). Isolated *E. coli* colonies from each plasmid preparation were grown overnight in LB Broth with Kanamycin (50 μg/mL) or Ampicillin (100 μg/mL) at 37 °C while shaking. After approximately 18 h, 2 mL of culture was processed using the PureLink HiPure Plasmid Miniprep Kit (Invitrogen). Plasmid concentration was quantified using a Nanodrop (Thermo Scientific).

### Mammalian cell culture

Neuro2a cells (ATCC, CCL-131) were cultured under standard conditions (5% CO_2_, 37 °C) in Modified Eagles Medium (EMEM, ATCC 30–2003) with 10% fetal bovine serum (FBS, Gibco) and 1% Penicillin/Streptomycin (Gibco). Cells underwent daily media changes and were passaged 1–2 times weekly. Only cells that had underwent less than 15 passages were used for this study.

### Plasmid transfection

Prior to transfection, cells were counted (Countess 3, Invitrogen) and plated to ensure 60–70% confluency on the day of transfection. Cells for live imaging were cultured in 24-well plates with coverslip inserts (229125, CELLTREAT) coated with Poly-D-Lysine (A3890401, Gibco). Cells that underwent fixation were cultured on 12 mm #1.5 coverslips (501929528, Electron Microscopy Sciences) coated with Poly-D-Lysine (A3890401, Gibco). Cells were transfected with Lipofectamine 3000 (L3000001, Invitrogen) following manufacturer’s protocol. Approximately 24-h post-transfection, cells were either collected for analysis or used for the differentiation assay outlined below.

### Differentiation

Research has shown that serum deprivation can induce Neuro2a cell differentiation characterized by the increase in both neurite number and length (Evangelopoulos et al., 2010, 2005; Lee et al., 2019; Martín et al., 2024). Evangelopoulos et al. (2005) established that culturing serum deprived Neuro2a cells in 0.1% bovine serum albumin (BSA) resulted in the activation of neural specific pathways. Serum deprivation was initially tested in our lab to confirm this pattern. Cells were imaged using phase contrast microscopy and compared using a Chi-square test. To determine Hsd17b7-GFP localization during differentiation, cells were washed with DPBS 24-h post-transfection and cultured in either complete medium (10% FBS) or differentiation medium (0% FBS, 0.1% BSA). Cells were incubated for 24 h, after which they were collected for staining and analysis. At least three replicates were completed for each experimental group.

### Live cell staining

Cells were stained 24-h post-transfection. ER staining was completed with ER-Tracker Red (E34250, Invitrogen,) while lysosomes were viewed using Lysoview 594 (70084, Biotium). Both stains were applied for 30 min at 37 °C using a 1 μM concentration of ER-Tracker Red and 1X working concentration of Lysoview 594 (in culture medium). Cells were imaged immediately after completion of dye incubation.

### Immunocytochemistry

The nuclear envelope was targeted with anti-Emerin (30853, Cell Signaling) and Lamin A/C (4777, Cell Signaling) primary antibodies. The Golgi apparatus was targeted with anti-RCAS1 primary antibody (12290, Cell Signaling) while dendrites were targeted with anti-MAP2 (4542, Cell Signaling) primary antibody. All antibodies were validated by Cell Signaling and Emerin and RCAS1 have been reported in Neuro2a cells (Koczok et al., 2019). Hsd17b7 was targeted with a polyclonal antibody (14854-1-AP, Proteintech), which had been knockout validated. Cells were briefly washed with phosphate buffered saline (PBS) and fixed using 4% paraformaldehyde for 10 min. Post-fixation, cells were washed again with PBS and underwent permeabilization in 0.1% TritonX-100 in PBS followed by blocking in 4% goat serum in PBS for 60 min at room temperature (RT). Cells were incubated overnight at 4 °C in diluted anti-Emerin antibody (1:400), anti-Hsd17b7 (1:500), anti-RCAS1 (1:400), or anti-Lamin A/C (1:100), or anti-MAP2 (1:500) in blocking solution. Cells were then washed with PBS and stained with either goat-anti-rabbit Alexa Fluor 594 (1:500, A11012, Invitrogen) or donkey-anti-mouse 594 (1:500, 715–585-151, Jackson ImmunoResearch Labs) for 60 min at RT in the dark. For Phalloidin counter staining, cells were washed with PBS and incubated in conjugated Phalloidin (00042, Biotium) for 20 min at RT in the dark. Cells were then washed with PBS and stained with Hoescht 3342 (62249, Thermo) for 5 min. Following Hoechst staining, cells were washed with PBS and coverslips were mounted with ProLong Diamond (P36965, Invitrogen). Slides were allowed to cure in the dark at RT for at least 24 h prior to imaging. Staining was completed for at least three experimental replicates for each antibody.

### Western blot

Transfection of both pCAG-GFP and N-terminal Hsd17b7 was done in 6-well culture dishes. Three wells were pooled for each, and whole cell lysates, along with cytosolic and nuclear fractions were prepared following modified REAP protocols (Suzuki et al., 2010) and stored at −80 °C. Total protein concentrations were quantified using the Pierce™ BCA Protein Assay (23227, Thermo). Samples were thawed on wet ice and centrifuged at 14,000 rpm for 10 min at 4 °C. 10 μL (lysate and cytosolic) and 5 μL (nuclear) were mixed with 5 μL of 4 × Laemmli buffer (161074, Bio-Rad) and diluted to 20 μL with DI water. Samples were boiled at 90 °C for 5 min, loaded onto 10% SDS-PAGE gels, and electrophoresed at 100 V for 10 min followed by 200 V for 45 min. Proteins were transferred to PVDF membranes (1620177, Bio-Rad) at 100 V for 120 min. Membranes were blocked in 5% milk/TBS-T and incubated with mouse anti-GFP (1:1000, sc-9996, Santa Cruze) or mouse anti-Lamin A/C (1:1000, 4777, Cell Signaling) or mouse anti-GAPDH (1:1000, sc-32233, Santa Cruze) for 48 h at 4 °C. After three 5-min washes in TBS-T, membranes were incubated with donkey anti-mouse HRP (1:20,000, 715–035-150, Jackson ImmunoResearch Labs) for 2 h at 4 °C. Detection was performed using Clarity ECL substrate (1705060, Bio-Rad) and imaged on a LI-COR C-DiGit.

### Imaging and analysis

Phase contrast images were collected with a Zeiss Axiovert. A1 (LD A-PLAN 40X) and Icm1 (Sony ICX 267 CCD, 1.4 Megapixel) monochrome camera and Zen Lite software (Version 3.10; Version 3.10; Carl Zeiss, Oberkochen, Germany). Immunofluorescence images were captured with an oil immersion 60X (PLAN-APO-OHR, NA = 1.5) objective on an Olympus FV4000 confocal microscope using FluoView software (Olympus). Regions of interest were selected using FIJI software (Schindelin et al., 2012). Neurite number was analyzed using NeuronJ: An ImageJ Plugin for Neurite Tracing and Quantification (RRID: SCR_002074). Images were prepared in Adobe Illustrator (Ver 28.2.1, Adobe).

## Results

### Comparison of GFP-tagged and endogenous Hsd17b7

**Figure 1:**
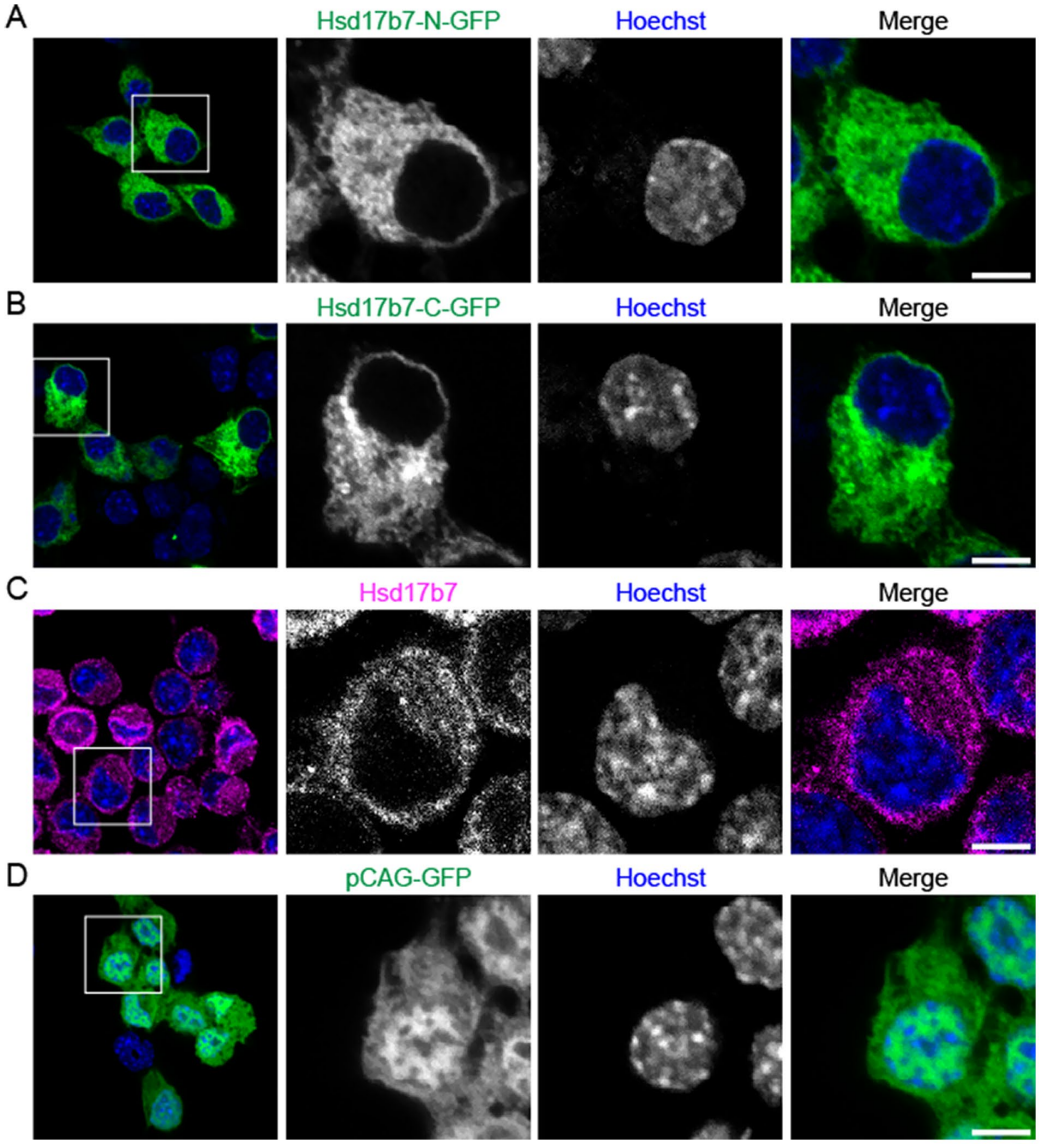
Confocal images comparing endogenous Hsd17b7 vs. GFP-tagged Hsd17b7 in undifferentiated Neuro2a cells. **(A,B)** Neuro2a cells transfected with either an N- or C- terminally tagged Hsd17b7 GFP construct and **(C)** Undifferentiated Neuro2a cells stained for Hsd17b7 antibody. **(D)** Undifferentiated Neuro2a cells were transfected with a pCAG-GFP construct. All groups were counterstained for the DNA marker Hoechst 334. Scale bars: 5 μM.

As a transmembrane protein, the C-terminus of Hsd17b7 resides on the cytosolic face while the N-terminus protrudes into the non-cytosolic face of the membrane (Tsachaki and Odermatt, 2019). Results from our transfection experiments in undifferentiated Neuro2a cells show that N-terminal tagged Hsd17b7-GFP signal localized around our DNA marker Hoechst (Figure 1A). While prior studies suggested that C-terminal tagged Hsd17b7 incurred cytosolic localization (Marijanovic et al., 2003); we observed a similar pattern to our N-terminal GFP construct (Figure 1B). In both cases, the GFP signal has an asymmetric accumulation on one side of the cell and a thin line surrounding the Hoescht DNA stain. This would suggest that the protein is likely localized within structures around the nucleus including the nuclear membrane and ER. When comparing the Hsd17b7-GFP patterns with endogenous Hsd17b7 protein, we did note increased signal intensity with the plasmid compared to our antibody stain (Figures 1A–C). This finding, however, was expected given the GFP-tagged plasmid’s increased expression of targeted proteins. Similar to our GFP-tagged Hsd17b7 results, antibody staining showed that the protein resided around our Hoechst DNA stain, without extensive staining within the nuclear compartment (Figure 1C). In comparison, our control pCAG-GFP plasmid, which has a generic eukaryotic promoter, produced non-specific signal in both the cytoplasm and within the nuclear compartment (Figure 1D). Moreover, when we co-stained Hsd17b7-GFP transfected cells with the Hsd17b7 antibody, both signals clearly overlapped suggesting that the plasmid was indeed representing the Hsd17b7 protein (Supplementary Figure 1). Given that N-terminal tags are more stable due to their luminal orientation (Tsachaki and Odermatt, 2019), the remainder of this study was completed using the N-terminal tagged Hsd17b7 plasmid.

### Hsd17b7 localizes to the ER and nuclear membrane in Neuro2a cells

**Figure 2:**
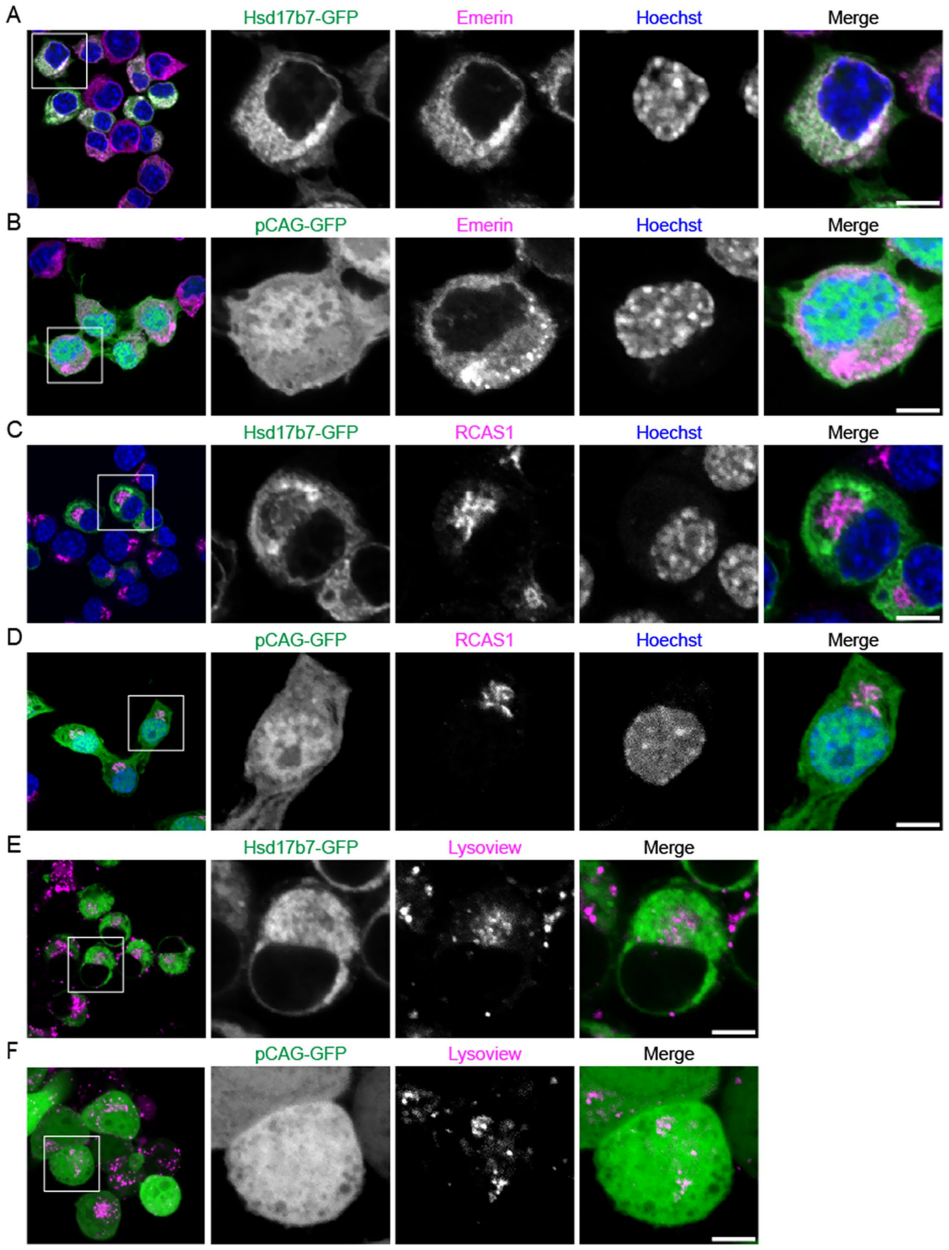
Confocal imaging of Hsd17b7 subcellular localization in undifferentiated Neuro2a cells. **(A–D)** Comparison of Hsd17b7-GFP and pCAG-GFP localization with the nuclear marker Emerin **(A,B)** and Golgi marker RCAS1 **(C,D)** in fixed cells. DNA counterstain was done with Hoechst 3342. **(E,F)** Comparison of Hsd17b7-GFP and pCAG-GFP localization in live cells using the lysosomal marker Lysoview. Scale bars: 5 μM.

Although it was postulated that this enzyme primarily localizes to the ER in mammalian cells (Tsachaki and Odermatt, 2019), it appears that like other post-squalene biosynthesis enzymes (Koczok et al., 2019), Hsd17b7 also associates with the nuclear membrane in undifferentiated Neuro2a cells (Figure 2A). Emerin is a protein that resides in the inner and outer nuclear membrane and peripheral ER (Salpingidou et al., 2007). Indeed, our Emerin immunostaining results aligned with recent findings in mouse primary neuron cultures, which highlights both the nuclear envelope as well at the ER (Xie et al., 2025; Figures 2A,B). Unlike pCAG-GFP, we observed an overlapping pattern between Emerin and Hsd17b7-GFP (Figures 2A,B). We further supported Hsd17b7 localization to the ER in live undifferentiated Neuro2a cells using ER Tracker (Supplementary Figure 2). Co-localization of Hsd17b7 with the nuclear envelope was further supported with subcellular fractionation of GFP transfected cells (Supplementary Figure 3). Fractionation results revealed that while much of the Hsd17b7-GFP protein appeared in the cytosolic fraction (aligning with ER localization), a small proportion also appears in the nuclear fraction. Staining with the nuclear envelope protein Lamin A/C also showed overlapping pattern with Hsd17b7-GFP further supporting that this protein associates with the nuclear membrane (Supplementary Figure 4).

In addition to the nuclear envelope and ER, we investigated whether Hsd17b7 localizes to other compartments that have been reported for enzymes in the post-squalene portion of cholesterol biosynthesis. Localization to the Golgi has been reported for the post-squalene cholesterol biosynthesis enzymes Nsdhl, which precedes Hsd17b7 in the pathway (Caldas, 2003). Using anti-RCAS1, a resident protein of the Golgi apparatus, we did not observe co-localization with Hsd17b7 (Figure 2C). These results were also reflected with our control pCAG-GFP plasmid (Figure 2D). Moreover, we did not observe co-localization for our Hsd17b7-GFP or pCAG-GFP plasmid in the lysosomal compartments of the cell (Figures 2E,F).

### Hsd17b7 is present in the neurites of differentiating Neuro2a cells

**Figure 3:**
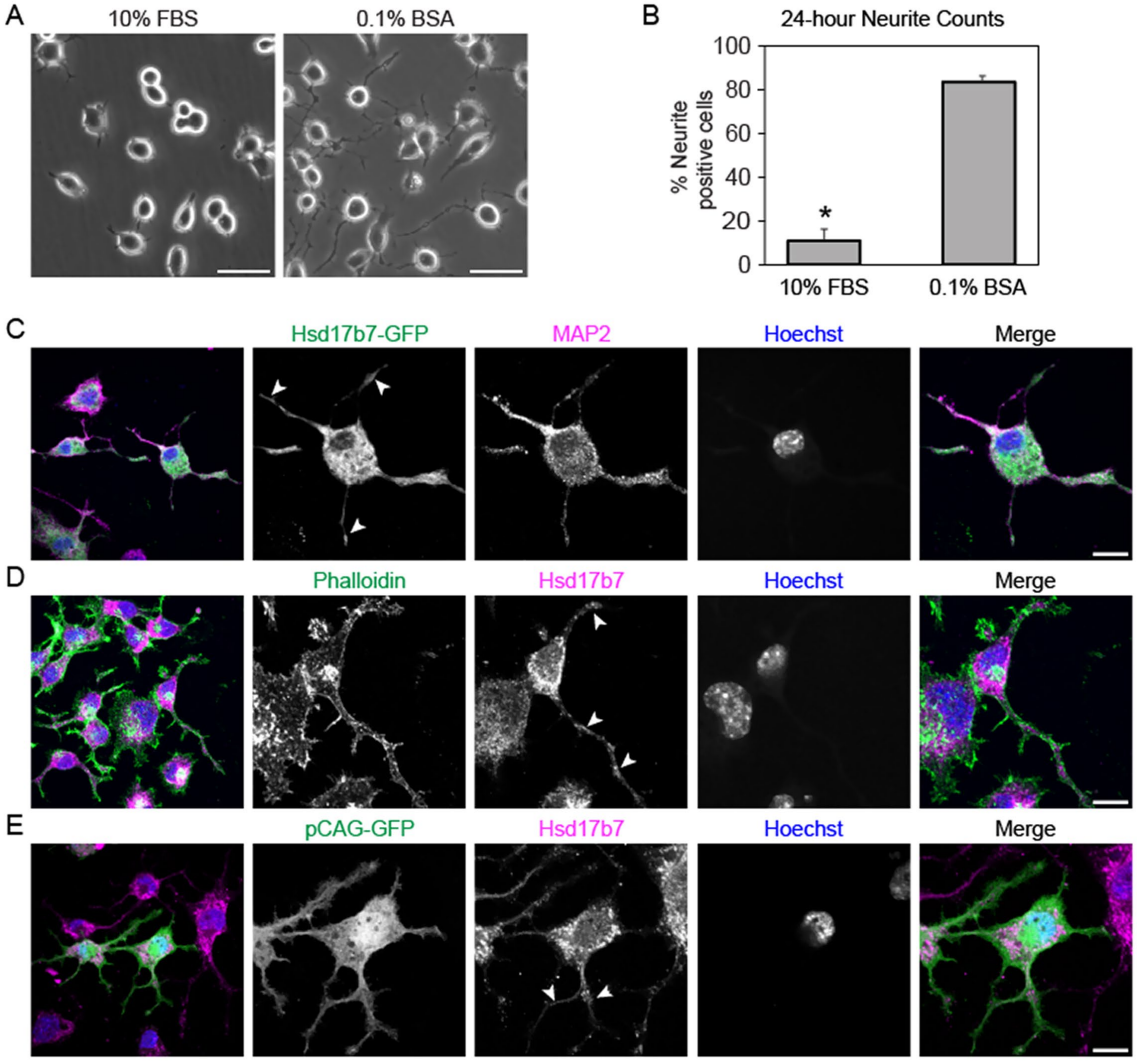
Phase contrast and confocal imaging analysis of Hsd17b7 localization in differentiating Neuro2a cells. **(A)** Phase contrast images of cells grown in media supplemented with 10%FBS or 0.1%BSA for 24-h. Scale bar: 50 μM **(B)** Quantification of neurite structures (n > 300 cells) in 0.1% BSA (85 ± 5%) compared to the 10% FBS control group (11 ± 2%). Values in the graph are given as average ± standard error. *denotes *p* < 0.001. **(C)** 24-h post-transfection with Hsd17b7-GFP Neuro2a cells were serum deprived for an additional 24 h and co-stained with MAP2 antibody. Arrows denote GFP signal in MAP2-positive structures. Scale bar: 5 μM. **(D)** After 24-h serum deprivation, cells were stained for anti-Hsd17b7 antibody and co-stained with conjugated Phalloidin-488. Arrows indicate Hsd17b7 signal in Phalloidin positive neurite structures. Scale bar: 5 μM. **(E)** 24-h post-transfection with pCAG-GFP Neuro2a cells were serum deprived for an additional 24 h and co-stained with Hsd17b7 antibody. Arrows denote antibody signal in pCAG-GFP positive neurite structures. Scale bar: 5 μM.

Given that a lack of Hsd17b7 is associated with precocious differentiation and abnormal neural membrane dynamics (Driver et al., 2016), we investigated protein localization during the process of neural differentiation. To model this, we conducted serum starvation 24 h post-transfection to induce the formation of neurites (Evangelopoulos et al., 2005). Indeed, after serum starvation we observed a significant increase (*p* < 0.001) in neurites for our serum depleted (0% FBS, 0.1% BSA) group compared to the 10% FBS control group (Figures 3A,B). When differentiation was conducted 24-h post-transfection, we observed Hsd17b7-GFP surrounding our DNA marker, Hoechst, in differentiated cells, similar to our observations in undifferentiated cells (Figure 3C). However, we also observed patterns of GFP within MAP2-positive neurites (Figure 3C). MAP2 highlights microtubules within dendrites, supporting the finding that Hsd17b7 can localize within these structures. Additionally, we observed similar results with antibody staining for endogenous Hsd17b7 and Phalloidin, which highlights filamentous actin (F-actin) within the cell (Figure 3D). F-actin can be present in both axons and dendrites, suggesting that Hsd17b7 may not be solely limited to dendrites during neural differentiation (Konietzny et al., 2017; Lavoie-Cardinal et al., 2020) (Figure 3D, arrows). We further confirmed the presence of Hsd17b7 in neurites by combining antibody staining with pCAG-GFP transfections (Figure 3E). Given the ubiquitous nature of the pCAG-GFP plasmid, this further highlighted neurite structures and supported presence of the Hsd17b7 protein outside of the cell body.

## Discussion

Overall, our study supports prior findings while expanding knowledge regarding the location of Hsd17b7 within mammalian neural cells. Given that protein localization can differ between mammalian cell types (Zhang et al., 2005), we aimed to establish the location of Hsd17b7 in a neural specific cell line. Studies have reported similarity in protein localization between Neuro2a cells and primary neurons, thus providing a model for localization during neural differentiation (Korutla et al., 2005; Maekawa et al., 2009). Moreover, while prior studies report localization in non-neural cells (Marijanovic et al., 2003; Stottmann et al., 2011), Hsd17b7 has not been fully characterized in a neural specific cell line. Therefore, we conducted fluorescent microscopy experiments in mouse Neuro2a cells. Derived from the neural crest (Huo et al., 2021), these cells have become standard for neural differentiation studies (Shea et al., 1985; Sugahara et al., 2019) with the ability to differentiate into neurons within 24 h of serum deprivation (Dos Santos et al., 2023; Evangelopoulos et al., 2010; Wu et al., 1998). While our data supports existing information regarding Hsd17b7 localization to the ER, we have also established protein localization with the nuclear membrane. In contrast, we did not observe localization with the Golgi apparatus or lysosomes. Additionally, we provide new insight into this protein’s location in developing neurons, with observations of localization within developing neurite structures. Further investigation as to how and why this protein is located within these structures is necessary to determine potential impacts on neural differentiation.

The use of GFP-tagged proteins has been shown to be an effective method for understanding protein localization in eukaryotic cells; however, location of the GFP-tag on the C- or N- terminus may impact localization (Stadler et al., 2013). When comparing the N- and C-terminal tagged constructs, our results do contrast to a prior study reporting that C-terminal tagged Hsd17b7 was restricted to cytosolic localization (Marijanovic et al., 2003). However, a more recent study that showed C-terminally tagged Hsd17b7 is capable of localizing to non-cytosolic regions (Tsachaki et al., 2015), which aligns with our findings. We did find similarities between the pattern of GFP-construct and our Hsd17b7 antibody with more intense signal generated from the GFP construct. This is expected, given the construct is generating a transient over-expression of the Hsd17b7 protein. Given the pattern consistency we observed, use of either antibody or GFP-plasmid are representative of Hsd17b7 localization in Neuro2a cells.

Our findings are supported by earlier studies in non-neural cells where this protein was identified in the ER (Marijanovic et al., 2003; Tsachaki et al., 2015; Tsachaki and Odermatt, 2019). The ER is the largest multifunctional organelle within the mammalian cell, possessing a diversity of roles including lipid modification, biosynthesis, and regulating intracellular calcium (Puhka et al., 2007; Sitia and Meldolesi, 1992). This localization also aligns with Hsd17b7’s role in sterol and steroid biosynthesis, which is known to occur in the ER (Sewer and Li, 2008).

Although it was postulated that this enzyme primarily localizes to the ER in mammalian cells (Marijanovic et al., 2003; Tsachaki and Odermatt, 2019), it appears that like other post-squalene biosynthesis enzymes (Koczok et al., 2019), Hsd17b7 also associates other compartments such as the nuclear membrane. Given that the outer ER membrane runs continuously with the nuclear membrane Hsd17b7 may have membrane mobility or a specific role within the nuclear membrane itself. Moreover, while other sterol desaturases in the cholesterol biosynthesis pathway, such as Nsdhl, have been reported in lysosomes (Caldas, 2003; Tsachaki and Odermatt, 2019), we did not observe localization of Hsd17b7 to these compartments. This is particularly interesting as Nsdhl participates in a neighboring step of post-squalene biosynthesis, and yet these two enzymes show diversity in location. This further emphasizes that these enzymes may possess unique roles within a neural cell.

While it has been reported that cholesterol biosynthesis does not occur in axons of primary neurons (Vance et al., 1994), other studies have shown evidence for the potential of cholesterol biosynthesis enzymes to exist within these structures. For example, data from a recent study shows the presence of Dhcr24 in the neurites of primary mouse neurons (Yavuz et al., 2021). Moreover, ablation of cholesterol biosynthesis has been connected to reduced neurite outgrowth in primary neurons (Fünfschilling et al., 2012). Loss of Hsd17b7 function has been associated with abnormal neurite structure during differentiation (Driver et al., 2016). Namely, primary neurons generated from Hsd17b7 mutant mice displayed significantly fewer lamellipodia/filopodia and showed accelerated maturation with formation of primary axons compared to controls (Driver et al., 2016). This is of particular interest, given we observed Hsd17b7 along presumptive axon structures within the Neuro2a cells. Given the association between Hsd17b7 and the ER, it is plausible that it could be within the neurite structure due to ER transport. Indeed, data has shown that ER in the soma runs continuously into both axons and dendrites of developing neurons with roles in axon development and synaptic connectivity (Ramirez et al., 2008; Yperman and Kuijpers, 2023). While the rough ER appears to be limited to soma and dendrite structures, the smooth ER extends the length of axons (Yperman and Kuijpers, 2023). It would be of interest to further investigate how Hsd17b7 is contributing to these structures, whether through cholesterol biosynthesis and/or steroid biogenesis pathways.

## Data Availability

The raw data supporting the conclusions of this article will be made available by the authors, without undue reservation.
