# Supplementary material for: Hsd17b7 undergoes dynamic subcellular localization during Neuro2a differentiation
Source: Front Mol Neurosci. 2026 Jan 15;18:1639803. doi: 10.3389/fnmol.2025.1639803 (PMC12852399; doi:10.3389/fnmol.2025.1639803)
Supplement: Supplementary file 1 [file Data_Sheet_1.pdf]

Bispo et al., Supplementary Material

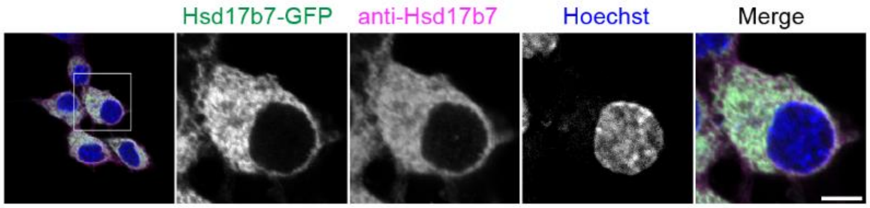

Supplementary Figure 1: Confocal imaging of GFP-tagged and endogenous Hsd17b7 in Neuro2a cells. Undifferentiated cells were transfected with N-terminal Hsd17b7-GFP and counterstained with anti-Hsd17b7 antibody and Hoechst 3342. Scale bar: 5μM.

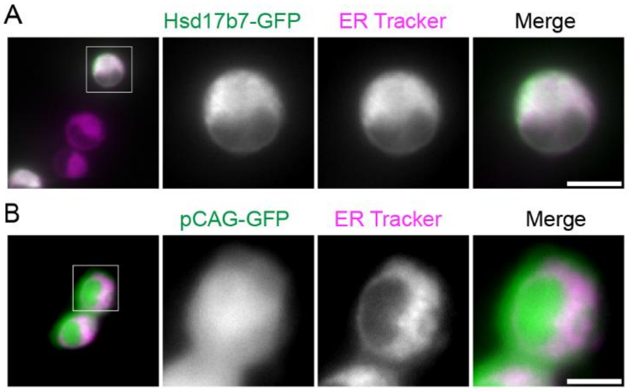

Supplementary Figure 2: Fluorescent imaging and localization in live undifferentiated Neuro2a cells. (A,B) 24-hours post-transfection cells with either N-terminal tagged Hsd17b7 GFP or pCAG-GFP cells were stained using the live cell marker ER Tracker Red. Scale bar: 10μM.

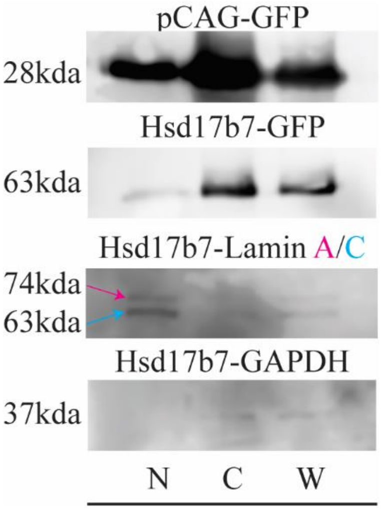

Supplementary Figure 3: Western blot of undifferentiated Neuro2a cellular fractions. GFP in nuclear fraction (N), cytosolic fraction (C), and whole cell (W) in pCAG-GFP and Hsd17b7-GFP cell line. GFP appeared at the expected size of 28kda in the pCAG-GFP samples. In contrast GFP was detected at 65 kda in the Hsd17b7-GFP samples, reflective of the combined size of the fusion protein (28kda+37kda). To confirm cell fractions, Hsd17b7-GFP cells were blotted for Lamin A/C (nuclear marker) and GAPDH (cytosolic marker). Lamin A/C was enriched in the nuclear fraction, followed by the whole cell fraction. Note, we did detect trace levels in the cytosolic fraction. Whereas GAPDH was only detected in cytosolic and whole cell fractions.

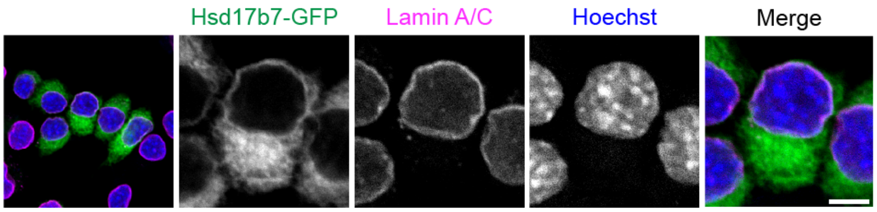

Supplementary Figure 4: Confocal imaging of the nuclear compartment in undifferentiated transfected Neuro2a cells. Cells were transfected with N-terminal Hsd17b7-GFP and counterstained with anti-Lamin A/C antibody and Hoechst 3342. Scale bar: 5μM.
